# Prevalence of food hypersensitivity in relation to IgE sensitisation to common food allergens among the general adult population in West Sweden

**DOI:** 10.1186/s13601-019-0261-z

**Published:** 2019-04-01

**Authors:** G. Rentzos, L. Johanson, Emma Goksör, E. Telemo, Bo Lundbäck, L. Ekerljung

**Affiliations:** 1 Krefting Research Centre, Institution for Medicine, Sahlgrenska Academy, University of Gothenburg, P.O Box 424, 405 30 Gothenburg, Sweden; 2 Department of Internal Medicine and Clinical Nutrition, Krefting Research Centre, Sahlgrenska Academy, University of Gothenburg, Gothenburg, Sweden; 3 Department of Pediatrics, University of Gothenburg, Queen Silvia Children’s Hospital, Gothenburg, Sweden; 4 Deptartment for Rheumatology and Inflammation Research, Sahlgrenska Academy, University of Gothenburg, Gothenburg, Sweden

**Keywords:** Food hypersensitivity, Food allergy, Sensitisation, IgE, Prevalence

## Abstract

**Background:**

The prevalence of self-experienced adverse reactions to foods seems to have an increasing trend in both adults and children. However, it is unclear if the prevalence of food hypersensitivity in the Swedish adult population is still rising, what symptoms are caused by different foods and which are the most common foods to which adults are more frequently IgE-sensitised.

**Methods:**

In a cross-sectional study based on questionnaire responses, interviews and clinical examinations as part of the West Sweden Asthma Study, 1042 subjects from the general population, 17–78 years of age, living in Västra Götaland, Sweden, were included. The subjects reported symptoms for 56 specified foods and blood samples were taken to examine the IgE-sensitisation pattern for 9 common foods.

**Results:**

Approximately 32% of adults reported food hypersensitivity, affecting mostly women and subjects less than 61 years old. The foods most often reported to cause adverse reactions were hazelnut (8.9%), apple (8.4%), milk (7.4%) and kiwi (7.3%). Less than one percent (0.9%) reported symptoms from ingestion of meat. Symptoms mostly affected the gastrointestinal tract (15%) and the skin (2.7%). Sixteen per cent were IgE-sensitised to common foods, most often to hazelnut (13.3%), peanut (4.9%) and almond (3.0%), while 5.9% reported symptoms and were IgE-sensitised to the same food, mainly to hazelnut (5.3%).

**Conclusions:**

The prevalence of self-reported food hypersensitivity in West Sweden indicates a rising trend. The correspondence between self-reported symptoms and IgE-sensitisation to foods is generally poor, except for hazelnut and almond which exhibit moderate or fair correlation.

## Background

It is well known that adverse reactions to foods are frequently reported and often have a negative impact on quality of life [1, 2]. In a recent food allergy review in Europe, self-reported food hypersensitivity in adults ranged from 3.5 to 20% with the lowest prevalence occurring in Eastern Europe and the highest in northern Europe [3]. An epidemiological study in the general population in Berlin showed that 35% reported symptoms, while only 3.6% were diagnosed with IgE-mediated food allergy [4]. In another recent Nordic study in the adult population, the prevalence of self-reported symptoms was 21% [5], while data from the Netherlands showed that the prevalence of self-reported symptoms varied between 39 and 54% [6]. Additionally, in northern China the prevalence of self-reported food allergy was estimated at 18% [7]. The prevalence of food hypersensitivity or adverse reactions to foods seems to be increasing among both adults and children even if long-term data is elusive [3, 8–19]. Data from these studies have been mainly provided by questionnaires to participants with suspected food intolerance with complementary sIgE tests, and by meta-analyses from epidemiological studies of food hypersensitivity. We have previously shown that adverse reactions to foods are associated with asthma [20], but it is unclear which foods cause most reactions and which organs are mostly affected in the Swedish population.

IgE reactivity is necessary but not sufficient to explain IgE-mediated allergy since underlying non-IgE mediated mechanism may account for a large proportion of the total number of food allergies [4]. The “golden standard” used to verify a food allergy is a double-blind, placebo-controlled food challenge (DBPCFC) [9, 21]. This is an expensive, time-consuming test and the patient may be at risk for anaphylactic reaction during the challenge. Other methods to diagnose food allergy include reports from self-experienced food allergy, which generally tend to overestimate the prevalence [3, 13, 22, 23], skin prick tests and specific IgE-tests. By combining the methods mentioned above, a better estimation of the true prevalence of food allergy can be achieved.

Recently, a newly discovered form of food allergy to mammalian (red) meat has been reported, which is clinically manifested by delayed allergic or anaphylactic reactions hours after the ingestion of red meat [24]. However, the data concerning the prevalence of allergy to red meat are still scarce.

The aims of this study were to:

- Assess the current prevalence of food hypersensitivity to different foods in western Sweden.
- Assess the prevalence of IgE-sensitisation to a number of common foods.
- Assess the self-reported symptoms in relation to IgE-sensitisation for different foods.
- Assess the distribution and type of symptoms for different foods between different organ systems.

## Methods

**Fig. 1:**
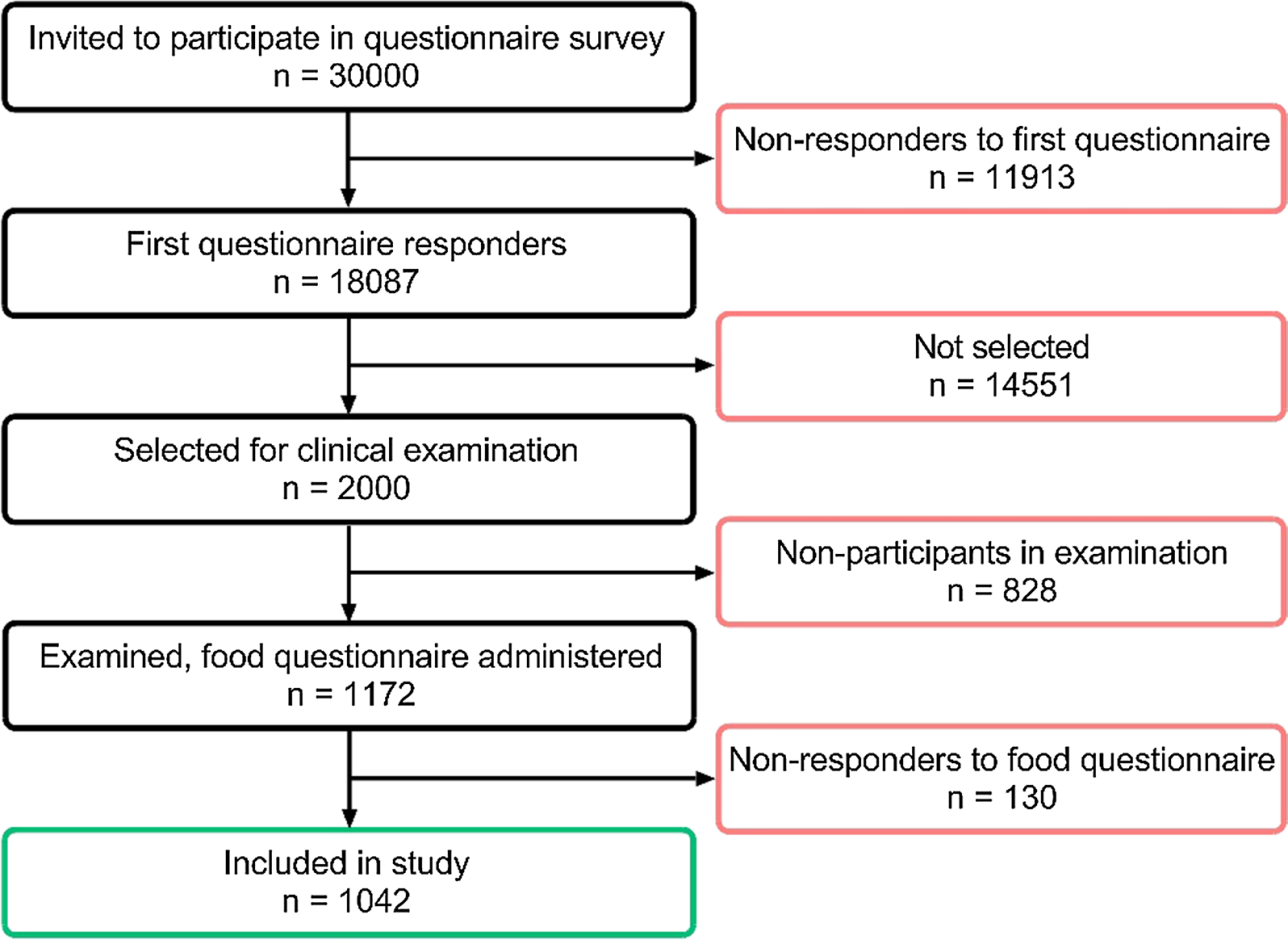
Flow-chart for the selection of participants to the study and number of responders, non-responders respectively

A postal questionnaire containing the Swedish OLIN and the international GA2LEN-questionnaires, described in detail before [25], was mailed to 30,000 randomly selected subjects, aged 16–75, living in the county of Västra Götaland, Sweden in 2008; 15,000 subjects in the urban area of Gothenburg and 15,000 in the remaining region. The response rate was 62%. A non-response study performed showed no differences in prevalence of symptoms or disease between responders and non-responders [26]. Out of the 18,087 responders, 2000 were randomly selected for clinical examination and interviews. Out of the 2000 invited subjects, 1172 participated in the clinical examinations and were given a second questionnaire with detailed questions on food hypersensitivity as well as other hypersensitivity symptoms, described in detail there [20]. Subjects who completed this questionnaire were included in the analyses, 1042 subjects in total. Some of the participants were on ongoing or intermittent medication for asthma, seasonal or other allergies. Further details concerning medication were not assessed in the present study. The demographic data for sex and age distribution are presented in Table 1. A flow chart of the study set up is presented in Fig. 1.

**Table 1:** Distribution of subjects included in the study according to sex and age

Total	Men	Women	Age				
			Mean age	17–30	31–45	46–60	≥ 61
1042 (100%)	480 (46.1%)	562 (53.9%)	51.0 ± 30.0	146 (14.0%)	255 (24.5%)	309 (29.7%)	332 (31.9%)

Blood samples for analysing the IgE-sensitisation profile of two ImmunoCAP allergen panels were collected; fx1 (peanut, hazel nut, brazil nut, almond, coconut) and fx5 (egg white, milk, fish, wheat, peanut, soy bean) and t3 (birch pollen) (Thermo Fischer Scientific, Uppsala, Sweden). All positive samples to a panel (> 0.35 kUA/l) were further analysed for each individual allergen included in that panel, according to the manufacturer’s instructions. Values > 0.35 kUA/l for the individual allergen indicated clinical IgE-sensitisation. The foods included in panels fx1 and fx5 were characterized as “common foods” since they comprise of some of the most frequent foods consumed on a daily basis in Sweden and other Western countries. Coconut was included in the IgE sensitisation tests but was not part of the questionnaire. Therefore it was not included in the calculations. This means that tests for IgE-sensitisation were determined for the above mentioned 9 out of the 56 foods included in the questionnaire. The combination of IgE-sensitisation and self-reported symptoms are referred to as “symptoms and IgE” in this study and may be used to estimate probable IgE-mediated food allergy for these 9 foods. For the remaining 47 foods, data indicating food hypersensitivity were available only from self-reported symptoms. For examining any correlation between self-reported symptoms and IgE-sensitisation, analyses for sex and age, and four different age groups were applied; 17–30; 31–45; 46–60; > 60 years of age.

### Data collection procedures

In order to estimate the prevalence of hypersensitivity to milk excluding lactose intolerance, complementary encoded fields for milk, sour milk and cheese were added. Participants with suspected lactose intolerance were excluded if they were stating the most relevant clinical symptoms for lactose intolerance, such as abdominal pain, flatulence, diarrhoea/loose stools or any other lactose intolerance symptom specified in the free text field. Likewise, participants with suspected gluten intolerance were excluded if they were most commonly stating relevant clinical symptoms, such as diarrhoea/loose stools, feeling of illness or tiredness, but also even abdominal pain, flatulence, hives or rashes, or any other gluten intolerance symptom specified in the free text field for wheat flour or any other flour.

All responses from the food hypersensitivity questionnaire, regarding reactions to different foods, were encoded for the different symptoms according to Table 2, as described before [20].

**Table 2:** Encoding of symptoms for self-reported hypersensitivity reactions in the food hypersensitivity questionnaire [20]

Code	Meaning
skin	Symptoms from the skin (urticaria, eczema, angioedema, flush, itching, tingling, skin pain, papules, redness etc.)
gi	Abdominal pain, diarrhoea, flatulence, reflux, vomiting, constipation, oral allergy syndrome (OAS) etc.
airup	Symptoms from the upper airways –nose (rhinitis, nasal congestion, nasal itching, sneezing, red nasal papules), eyes etc.
airlo	Lower airways—respiratory symptoms (heavy breathing, difficulty getting air, wheezing, cough, chest pressure, bronchospasm, hoarseness, mucus in the throat)
circ	Palpation, fainting, dizziness
cns	Headache, confusion
ana	Anaphylactic reactions
gen	General symptoms such as tiredness, feeling ill
oth	Other (for example ear itching, gallstone)
not	Do not eat
unk	Unknown, uncertain whether hypersensitive or not

### Statistics

The statistical analyses were performed using SPSS 22.0.

Chi-squared test was used to compare the prevalence of adverse food reactions and IgE-sensitisation between groups, *p* values were calculated using Fisher’s two tailed exact test, and *p* values < 0.05 were considered statistically significant. Comparisons between reported symptoms and positive IgE-sensitisation were performed using the kappa-coefficient and were interpreted using the following definitions: below 0.2 slight or poor agreement; 0.21–0.4 fair agreement; 0.41–0.6 moderate agreement; 0.61–0.8 substantial agreement and 0.81–1 almost perfect agreement [27].

## Results

### Prevalence of self-reported food hypersensitivity in adults in the general population in Sweden

The prevalence of self-reported food hypersensitivity to any of the 56 foods in the questionnaire was 32.5% (95% CI 29.6–35.4) and was more frequent among women in comparison to men (39.1% vs. 24.1%, *p* < 0.001). Adults over 60 years of age reported the least hypersensitivity, and even less in those over 70. The foods most frequently reported to cause hypersensitivity reactions were hazelnut (8.9%), followed by apple (8.4%), milk (7.4%) and kiwi (7.3%). When excluding patients with suspected lactose intolerance symptoms, the prevalence of self-reported milk hypersensitivity was estimated to 1.1%. When excluding patients with suspected gluten intolerance symptoms, the prevalence of self-reported adverse reactions to wheat was estimated to 0.1%, compared to 1.6% including gluten intolerance related symptoms. Hypersensitivity reactions to red meat were reported by 0.9% of participants, out of which pork accounted for 0.6% and beef for 0.4%. Details concerning self-reported symptoms from the different foods are presented in Table 3.

**Table 3:** Prevalence of self-reported symptoms for different foods, according to sex and age distribution (%)

Food	Total (95% CI)	Men	Women	*p* value	Age				
					17–30	31–45	46–60	> 60	*p*_test for trends_
Any of the below	32.5 (29.6–35.4)	24.7	39.1	*< 0.001*	38.6	40.6	36.9	19.1	*< 0.001*
Additives	0.4 (0.0–0.8)	0.0	0.7	0.128	0.0	0.0	1.0	0.3	0.373
Almond	3.7 (2.5–4.8)	2.1	5.1	*0.013*	4.1	6.3	4.2	0.9	*0.007*
Anise/caraway	0.2 (–0.1–0.5)	0.0	0.4	0.502	0.0	0.4	0.3	0.0	0.691
Apple	8.4 (6.7–10.1)	6.3	10.3	*0.024*	11.8	12.6	9.1	3.0	*< 0.001*
Apricot	1.7 (0.9–2.5)	1.5	2.0	0.637	1.4	2.7	1.6	1.2	0.443
Avocado	0.8 (0.2–1.3)	0.8	0.7	1.000	0.7	1.6	1.0	0.0	0.139
Banana	1.3 (0.6–2.0)	0.8	1.8	0.280	1.4	2.7	1.3	0.3	0.066
Bean	1.8 (1.0–2.6)	0.8	2.7	*0.035*	2.1	2.0	2.6	0.9	0.372
Beef	0.4 (0.0–0.8)	0.2	0.5	0.629	0.0	0.0	1.3	0.0	0.693
Brazil nut	4.2 (3.0–5.4)	2.9	5.3	0.085	4.1	6.0	5.6	1.5	0.053
Camomile	0.6 (0.1–1.0)	0.2	0.9	0.226	1.4	0.8	0.6	0.0	0.061
Carrot	3.1 (2.0–4.1)	2.1	3.9	0.105	3.4	6.3	2.9	0.6	*0.003*
Cayenne/red pepper	1.6 (0.9–2.4)	1.5	1.8	0.808	0.7	2.0	2.3	1.2	0.899
Cheese	1.8 (1.0–2.6)	2.7	1.1	0.062	2.8	1.2	2.6	1.2	0.484
Cheese^a^	0.8 (0.0–0.0)	1.5	0.2	*0.027*	1.4	0.0	1.3	0.6	0.893
Chestnut	0.5 (0.1–0.9)	0.4	0.5	1.000	0.7	1.2	0.3	0.0	0.088
Chicken	0.1 (− 0.1–0.3)	0.0	0.2	1.000	0.0	0.0	0.3	0.0	0.845
Celery	0.3 (0.0–0.6)	0.2	0.4	1.000	0.7	0.4	0.3	0.0	0.186
Cherry	3.1 (2.0–4.1)	2.9	3.2	0.858	3.4	5.5	3.3	0.9	*0.013*
Chili/tabasco	2.2 (1.3–3.1)	1.3	3.0	0.058	1.4	2.8	3.2	1.2	0.652
Chocolate	1.6 (0.9–2.4)	1.3	2.0	0.465	1.4	1.6	1.6	1.8	0.718
Coriander	0.1 (− 0.1–0.3)	0.0	0.2	1.000	0.0	0.0	0.3	0.0	0.842
Curry	1.0 (0.4–1.6)	0.8	1.1	0.760	0.7	0.8	1.6	0.6	0.976
Dried fruit	0.3 (0.0–0.6)	0.0	0.5	0.254	0.0	0.4	0.6	0.0	0.834
Egg	1.3 (0.6–2.0)	0.6	2.0	0.102	2.1	1.6	1.0	1.2	0.422
Fish	0.3 (0.0–0.6)	0.2	0.4	1.000	0.0	0.4	0.7	0.0	0.833
Flour (non wheat)	0.7 (0.2–1.2)	0.4	0.9	0.461	3.4	0.0	0.6	0.0	*0.002*
Flour (non wheat)^b^	0.0 (0.0–0.0)	0.0	0.0	–	0.0	0.0	0.0	0.0	–
Flour (wheat)	1.6 (0.9–2.4)	1.0	2.1	0.221	2.1	2.0	1.9	0.9	0.287
Flour (wheat)^b^	0.1 (− 0.1–0.3)	0.0	0.2	1.000	0.0	0.0	0.3	0.0	0.847
Fried/fat food	3.7 (2.5–4.8)	1.7	5.4	*0.001*	6.2	3.5	4.2	2.2	0.060
Hazelnut	8.9 (7.1–10.6)	6.7	10.8	0.280	6.9	12.3	11.0	5.1	0.109
Kiwi	7.3 (5.7–8.8)	2.9	11.0	*< 0.001*	10.5	11.0	6.9	3.3	*< 0.001*
Lingonberry	0.1 (− 0.1–0.3)	0.0	0.2	1.000	0.0	0.0	0.0	0.3	0.245
Melon	0.4 (0.0–0.8)	0.4	0.4	1.000	0.7	0.8	0.3	0.0	0.126
Milk	7.4 (5.8–9.0)	4.8	9.6	*0.004*	11.7	9.8	9.5	1.5	*< 0.001*
Milk^a^	1.1 (0.4–1.7)	1.1	1.1	1.000	3.5	0.4	1.0	0.6	0.050
Nectarine	2.4 (1.5–3.3)	1.3	3.4	*0.026*	1.4	5.1	2.9	0.3	*0.034*
Others	3.6 (2.5–4.8)	2.5	4.6	0.071	2.7	5.9	3.2	2.7	0.328
Orange	2.9 (1.9–3.9)	1.0	4.5	*0.001*	1.4	2.1	3.6	1.2	0.224
Parsley	0.3 (0.0–0.6)	0.2	0.4	1.000	0.7	0.8	0.0	0.0	0.060
Pea	0.7 (0.2–1.2)	0.2	1.1	0.132	1.4	0.8	1.0	0.0	0.098
Peach	3.2 (2.1–4.3)	2.9	3.4	0.725	4.2	5.5	2.0	2.1	*0.036*
Peanut	3.5 (2.4–4.6)	1.9	4.9	*0.01*	6.2	2.0	4.9	2.1	0.167
Pear	4.0 (2.8–5.2)	3.3	4.7	0.344	6.2	5.1	4.2	2.1	*0.021*
Plum	3.0 (2.0–4.0)	3.6	2.5	0.363	4.2	4.7	2.6	1.5	*0.026*
Poppy seed	0.1 (− 0.1–0.3)	0.0	0.2	1.000	0.0	0.4	0.0	0.0	0.445
Pork	0.6 (0.1–1.0)	0.0	1.1	*0.034*	0.7	0.8	1.0	0.0	0.274
Potato	1.6 (0.9–2.4)	1.0	2.1	0.221	2.1	3.9	1.3	0.0	*0.003*
Red meat	0.9 (0.3–1.4)	0.2	1.4	*0.043*	0.7	0.8	2.0	0.0	0.488
Salami	0.5 (0.1–0.9)	0.2	0.7	0.380	0.7	0.4	0.7	0.3	0.680
Sesame seed	0.1 (− 0.1–0.3)	0.0	0.2	1.000	0.0	0.0	0.0	0.3	0.246
Shellfish	3.5 (2.4–4.6)	2.5	4.3	0.127	2.1	4.3	3.6	3.4	0.789
Sour milk/yogurt	3.6 (2.4–4.7)	2.5	4.5	0.095	6.9	5.5	3.6	0.6	*< 0.001*
Sour milk/yogurt^a^	0.6 (0.1–1.1)	0.6	0.5	1.000	2.1	0.0	1.0	0.0	0.059
Soy	0.3 (0.0–0.6)	0.0	0.5	0.254	0.7	0.4	0.3	0.0	0.183
Strawberry	2.7 (1.7–3.7)	1.0	4.1	*0.003*	2.1	4.7	3.2	0.9	0.091
Sunflower seed	0.1 (− 0.1–0.3)	0.0	0.2	1.000	0.0	0.0	0.3	0.0	0.843
Sweet pepper	2.1 (1.2–3.0)	0.6	3.4	*0.002*	2.1	2.4	3.2	0.9	0.363
Tomato	2.1 (1.2–3.0)	0.6	3.4	*0.002*	4.8	2.8	2.6	0.0	*0.001*
Walnut	4.9 (3.5–6.2)	3.8	5.8	0.147	6.3	6.7	5.2	2.4	*0.020*
Wine/beer	4.6 (3.4–5.9)	3.1	5.9	*0.037*	4.8	7.1	5.2	2.1	*0.034*

Significant *p* values are marked with italic (*p* < 0.05)

^a^Lactose intolerance symptoms excluded

^b^Gluten intolerance symptoms excluded

### Prevalence of IgE sensitisation alone and in combination with self-reported symptoms to the most common foods

**Fig. 2:**
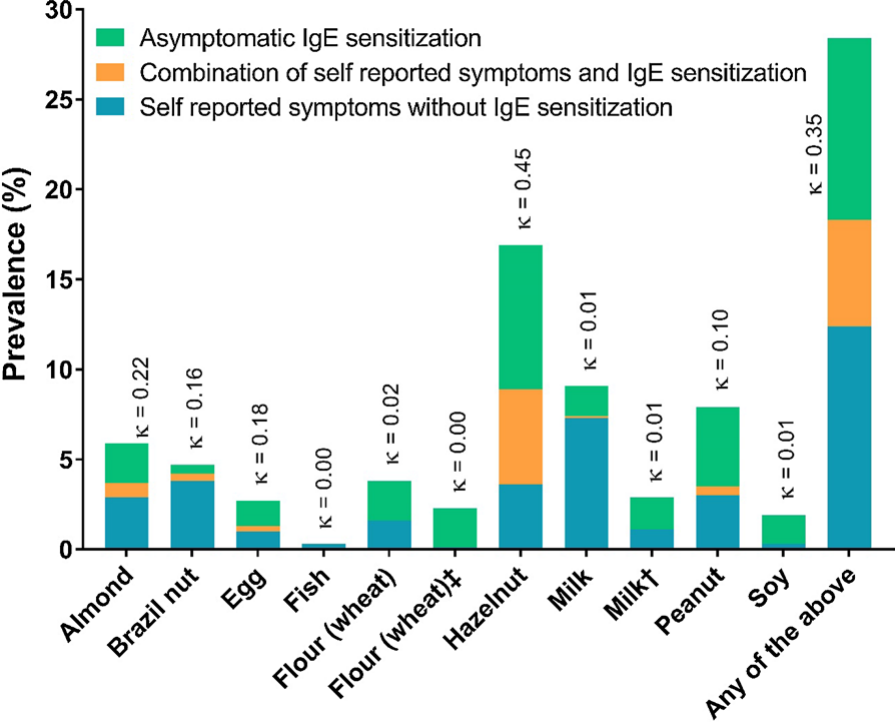
The prevalence of subjects with self-reported symptoms, IgE-sensitisation, and combination of self-reported symptoms with IgE sensitisation for the different common foods. Correspondence between symptoms and IgE-sensitisation using kappa-coefficients (κ < 0.2 for slight or poor agreement; κ 0.21–0.4 for fair agreement; κ 0.41–0.6 for moderate agreement; κ 0.61–0.8 for substantial agreement and κ 0.81–1 for almost perfect agreement). Dagger: Lactose intolerance symptoms excluded. Double dagger: Gluten intolerance symptoms excluded

Sixteen per cent of the subjects (95% CI 13.8–18.3) were IgE-sensitised to any of the 9 common foods, most often to hazelnut (13.3%) and peanut (4.9%). Subjects under 46 years of age were IgE-sensitised more often than older subjects. In addition, 5.9% (95% CI 4.5–7.4) were both IgE-sensitised and reported symptoms for any of the tested foods, most often to hazelnut (5.3%, 59.6% of those reporting symptoms from hazelnut). It is worth mentioning that 14.6% of the subjects were IgE-sensitised to birch pollen, one of the most common aeroallergens in the Swedish population. None of the subjects who reported symptoms from fish, soy or wheat was at the same time IgE-sensitised to these foods. Details are presented in Table 4. The correlation between self-reported symptoms and IgE-sensitisation for hazelnut was moderate (0.45), for almond was weak (0.22), whereas for the other foods it was poor, see also Fig. 2.

**Table 4:** Prevalence of positive IgE tests for the most common foods, in combination with self-reported symptoms for the different foods, according to sex and age distribution (%)

Food	Type	Total (95% CI)	Men	Women	*p* value	Age				
						17–30	31–45	46–60	> 60	*p*_test for trends_
Any of the below	IgE	16.0 (13.8–18.3)	18.4	13.9	0.058	24.6	20.6	13.7	10.8	*< 0.001*
	Symptoms and IgE	5.9 (4.5–7.4)	5.1	6.7	0.29	5.6	10.7	6.7	1.8	*0.002*
Almond	IgE	3.0 (1.9–4.0)	3.4	2.6	0.46	3.5	3.6	3.8	1.5	0.17
	Symptoms and IgE	0.8 (0.2–1.3)	0.4	1.1	0.30	0.7	2.0	0.7	0.0	0.067
Brazil nut	IgE	0.9 (0.3–1.5)	0.9	0.9	1.0	1.4	1.2	0.7	0.6	0..31
	Symptoms and IgE	0.4 (0.0–0.8)	0.4	0.4	1.0	0.7	1.2	0.0	0.0	*0.044*
Egg	IgE	1.7 (0.9–2.5)	1.5	1.9	0.81	2.1	0.0	1.7	2.8	0.13
	Symptoms and IgE	0.3 (0.0–0.6)	0.2	0.4	1.0	0.0	0.0	0.3	0.6	0.15
Fish	IgE	0.0 (0.0–0.0)	0.0	0.0	–	0.0	0.0	0.0	0.0	–
	Symptoms and IgE	0.0 (0.0–0.0)	0.0	0.0	–	0.0	0.0	0.0	0.0	–
Flour (wheat)	IgE	2.2 (1.3–3.1)	3.0	1.5	0.13	1.4	2.0	3.4	1.5	0.91
	Symptoms and IgE	0.0 (0.0–0.0)	0.0	0.0	–	0.0	0.0	0.0	0.0	–
Flour (wheat)^b^	Symptoms and IgE	0.0 (0.0–0.0)	0.0	0.0	–	0.0	0.0	0.0	0.0	–
Hazelnut	IgE	13.3 (11.2–15.4)	15.0	11.9	0.16	22.5	19.8	11.0	6.5	*< 0.001*
	Symptoms and IgE	5.3 (4.0–6.7)	4.8	5.7	0.58	4.1	9.9	6.2	1.5	*0.006*
Milk	IgE	1.8 (1.0–2.6)	2.1	1.5	0.48	1.4	1.6	1.7	2.2	0.54
	Symptoms and IgE	0.1 (− 0.1–0.3)	0.0	0.2	1.0	0.0	0.4	0.0	0.0	0.44
Milk^a^	Symptoms and IgE	0.0 (0.0–0.0)	0.0	0.0	–	0.0	0.0	0.0	0.0	–
Peanut	IgE	4.9 (3.5–6.2)	6.6	3.3	*0.018*	7.0	5.6	5.5	2.8	*0.037*
	Symptoms and IgE	0.5 (0.1–0.9)	0.2	0.7	0.38	2.1	0.4	0.3	0.0	*0.010*
Soy	IgE	1.6 (0.8–2.4)	2.1	1.1	0.22	1.4	1.6	2.4	0.9	0.68
	Symptoms and IgE	0.0 (0.0–0.0)	0.0	0.0	–	0.0	0.0	0.0	0.0	–

Significant *p* values are marked with italic (*p* < 0.05)

^a^Lactose intolerance symptoms excluded

^b^Gluten intolerance symptoms excluded

### Prevalence of reported symptoms from different organ systems

The subjects reported more often symptoms from the gastrointestinal tract (GI-tract) (15%) followed by the skin (2.7%). For the GI-tract, hazelnut was most often reported to cause symptoms (6.5%), followed by milk (6.2%, including symptoms due to suspected lactose intolerance), almond (2.7%), brazil nut (2.7%) and peanut (2.1%). Hazelnut (4.2%) was also the food that most subjects were IgE-sensitised to in the blood tests along with the reported GI-tract symptoms. Details are presented in Table 5.

**Table 5:** The percentage of subjects with self-reported symptoms and IgE-sensitization for common foods, divided into symptoms from the gastrointestinal tract (GI-tract), skin, lower and upper airways, anaphylaxis and other symptoms (as described and encoded in Table 2)

	GI (%)	Skin (%)	Lower airways (%)	Upper airways (%)	Anaphy-laxis (%)	Other (%)	Total (%)
*Total*							
Symptoms	15.0	2.7	1.6	0.6	0.1	1.6	18.0
Symptoms and IgE	4.9	1.2	0.5	0.3	0.1	0.2	5.9
*Almond*							
Symptoms	2.7	0.7	0.4	0.1	#	0.1	4.0
Symptoms and IgE	0.7	0.1	#	#	#	#	0.8
*Brazil nut*							
Symptoms	2.7	1.1	0.6	0.1	0.1	0.1	4.7
Symptoms and IgE	0.2	0.2	0.2	#	0.1	#	0.7
*Egg*							
Symptoms	1.2	#	0.1	#	#	#	1.3
Symptoms and IgE	0.2	#	0.1	#	#	#	0.3
*Fish*							
Symptoms	0.2	0.1	#	#	#	#	0.3
Symptoms and IgE	#	#	#	#	#	#	#
*Hazel nut*							
Symptoms	6.5	1.7	0.7	0.4	0.1	0.2	9.6
Symptoms and IgE	4.2	0.9	0.4	0.3	0.1	0.1	5.9
*Milk*							
Symptoms	6.2	0.1	0.2	#	#	0.97	7.5
Symptoms and IgE	0.1	#	#	#	#	#	0.1
*Milk* ^*a*^							
Symptoms	0.1	0.2	0.3	#	#	0.4	0.99
Symptoms and IgE	#	#	#	#	#	#	#
*Peanut*							
Symptoms	2.1	1.1	0.5	#	#	0.1	3.8
Symptoms and IgE	0.5	#	#	#	#	#	0.5
*Soy*							
Symptoms	0.3	#	#	#	#	#	0.3
Symptoms and IgE	#	#	#	#	#	#	#
*Wheat*							
Symptoms	1.4	#	0.1	#	#	0.1	1.6
Symptoms and IgE	#	#	#	#	#	#	#
*Wheat* ^*b*^							
Symptoms	#	#	0.1	#	#	#	0.1
Symptoms and IgE	#	#	#	#	#	#	#

#: < 0.01%

^a^Lactose intolerance symptoms excluded

^b^Gluten intolerance symptoms excluded

## Discussion

In the present study, approximately one out of three adults (32.5%) reported hypersensitivity reactions to foods, mostly to hazelnut. Approximately one out of six were IgE-sensitised, mostly to hazelnut, but the correlation between self-reported symptoms and IgE-sensitisation was generally poor, except for hazelnut where it was moderate and for almond where it was fair. Most participants reported symptoms from the GI-tract. Approximately 6% of adults reported food-related symptoms and were also IgE-sensitised to any of these nine common foods, indicating food allergy. Less than 1% of reported symptoms arose from ingestion of red meat. IgE-sensitisation and reports for food hypersensitivity declined with increasing age.

In the present study, the prevalence of self-reported food hypersensitivity for the different foods, IgE-sensitisation and combination of both for the most common foods are in line or slightly higher than previous studies [3–5, 23, 28]. In those studies, the prevalence of IgE-sensitisation was 4–16%, while the prevalence of the combination of IgE-sensitisation and self-reported symptoms, indicating IgE-mediated food allergy, were 2.2–3%. It was only Zuberbier et al. though who performed double-blind-placebo-controlled food challenges, if food intolerance could not be ruled out by the patient history [4].

Interestingly in the present study, the prevalence of self-reported food hypersensitivity was similar in age groups 17–30, 31–45 and 46–60, and decreased after 60 years of age. Similar results are reported recently in Georgia [29]. The same pattern was also observed when combining self-reported food-related symptoms with positive IgE-tests to common foods. When looking only at the IgE-sensitisation profile for the most common foods, a lower prevalence of IgE-sensitisation was noticed for the two oldest age groups (46–60 and > 60 years of age) compared to younger patients (17–30 and 31–45 years of age). This is partly in line with data from a previous review [3], where subjects > 60 years of age in Europe reported less symptoms, but were not less IgE-sensitised compared to younger subjects. In the same review, the prevalence of self-reported symptoms combined with positive IgE-tests was more or less the same for the different age groups, however the age group range was wider. In a recent Swedish study, IgE-sensitisation to specific foods decreased with increasing age when following a population over 9 years [5]. Further research is needed though to examine this issue in more detail.

Seven out of ten foods that have the most often reported adverse reactions, are also related to birch pollen (almond, apple, brazil nut, hazelnut, kiwi, pear and walnut). This may be explained by the fact that sensitisation to birch pollen is common in Sweden, which is in line with the results of this study showing that 14.6% of the subjects are IgE-sensitised to birch pollen [30, 31]. Moreover, many subjects also reported adverse reactions to milk (7.4%), including subjects with suspicious lactose intolerance, which is well in accordance with the prevalence of the lactose intolerance genotype in Sweden, 6–9% [32]. Hazelnut was the food that most subjects were IgE-sensitised to, followed by peanut and almond. This is in line with previous studies [29, 33], and may indicate that nuts (or nut-like) foods are highly IgE-sensitizing. Hazelnut was also the food with the best correlation when combining both symptoms and IgE-sensitisation, followed by other birch related foods in the Swedish population. It is also interesting that among the tested subjects, no one was IgE-sensitised to fish, which is in line with the results from previous studies showing very low prevalence of fish allergy in the adult population in Sweden (< 0.3%) and in North America (< 0.6%) [34–36].

It is well known that self-reported symptoms greatly overestimate true allergy [3, 13, 22, 23]. An example from this study is the low correlation between self-reported symptoms and IgE-sensitisation for soy, milk and wheat, and the non-existing correlation when excluding symptoms possibly caused by lactose and gluten intolerance (Fig. 2). For these foods, the correlation was even lower than random, which could give rise to considerations for probable tolerance development with increasing age in the adult population [37]. Only hazelnut reached moderate correlation, which may be explained by the strong association to birch pollen allergy, which is very common in Sweden [38].

The prevalence of hypersensitivity to any red meat was found to be 0.9%. Since this type of hypersensitivity reaction is relatively rare, there are very few studies examining it. Schäfer et al. found that 0.6% reported symptoms from meat in Germany [39]. The sensitisation route for this allergy includes tick-bites, where saliva from ticks may contain carbohydrate α-1,3-galactose (α-gal), which is also present in red meat. This may induce IgE antibodies to beef and/or pork meat. In the present study, more subjects reported symptoms to pork than to beef, and only one person (0.1%) reported symptoms from both. For pork, only females, and for beef only subjects aged 46–60 reported symptoms. The relatively older age could possibly be related to a higher risk of having had tick bite(s) earlier [40]. However, in this study the relation to tick bites could not be further assessed.

Many subjects also reported hypersensitivity reactions to alcohol (wine/beer, 4.6%), fried/fat food (3.7%), spices such as chili/tabasco (2.2%), potato (1.6%), curry (1%) and cereals other than wheat (0.7%), which may indicate IBS-like symptoms. It has previously been shown that subjects with IBS experience worsening of their symptoms after the consumption of spicy foods or foods rich in carbohydrates and fat [41].

In the current study, the absolute majority of reported symptoms were related to the GI-tract, which was also true for subjects having positive IgE-tests for the common foods. These results are in line with previous studies even though the reported prevalence varies greatly [42–44].

This study has some limitations that should be taken into consideration. The participation rate in the clinical examinations was 59% and the response rate for the food questionnaire was 89%. As expected, self-reported food intolerance yields a much higher prevalence compared to the IgE-sensitisation tests. It would have been advantageous to have a more extended IgE-sensitisation tests, including tests for food allergen components, and to retrieve more specific information about lactose and gluten intolerance in the studied subjects. It would have been interesting if there was available information about how many subjects with self-reported symptoms from red meat has been exposed to tick-bites including analyses from IgE-tests to α-gal. Despite these limitations, the large number of participants in the study makes the findings reliable and fascinating, as there are very few studies to date having examined the relation between food hypersensitivity and IgE-sensitisation to the most common foods indicating food allergy in the Swedish adult population.

In conclusion, every third adult reported food hypersensitivity, indicating a tendency for a rising trend compared with previous studies. Most subjects report symptoms from the GI-tract, and IgE-sensitisation and food hypersensitivity declined with increasing age. Hazelnut was the food most often reported to cause symptoms and showed the highest correlation with a positive IgE-tests. In general a large discrepancy between IgE sensitisation and self-reported symptoms was noticed, where only 6% of the subjects that reported food-related symptoms also were IgE-sensitised to the food causing their symptoms.
